# Does personality affect premating isolation between locally-adapted populations?

**DOI:** 10.1186/s12862-016-0712-2

**Published:** 2016-06-23

**Authors:** Carolin Sommer-Trembo, David Bierbach, Lenin Arias-Rodriguez, Yesim Verel, Jonas Jourdan, Claudia Zimmer, Rüdiger Riesch, Bruno Streit, Martin Plath

**Affiliations:** College of Animal Science and Technology, Northwest A&F University, Yangling, 712100 People’s Republic of China; Department of Ecology and Evolution, J.W. Goethe University Frankfurt, Max-von-Laue-Straße 13, 60438 Frankfurt am Main, Germany; Department of Biology and Ecology of Fishes, Leibniz-Institute of Freshwater Ecology and Inland Fisheries, Müggelseedamm 310, 12587 Berlin, Germany; División Académica de Ciencias Biológicas, Universidad Juárez Autónoma de Tabasco, Villahermosa, Tabasco CP. 86150 Mexico; Biodiversity and Climate Research Centre (BiK-F), Senckenberganlage 25, 60325 Frankfurt am Main, Germany; School of Biological Sciences, Royal Holloway, University of London, Egham Hill, Egham, TW20 0EX UK

**Keywords:** Premating isolation, Animal personality, Ecological speciation, Mate choice, Local adaptation, Assortative mating

## Abstract

**Background:**

One aspect of premating isolation between diverging, locally-adapted population pairs is female mate choice for resident over alien male phenotypes. Mating preferences often show considerable individual variation, and whether or not certain individuals are more likely to contribute to population interbreeding remains to be studied. In the *Poecilia mexicana*-species complex different ecotypes have adapted to hydrogen sulfide (H_2_S)-toxic springs, and females from adjacent non-sulfidic habitats prefer resident over sulfide-adapted males. We asked if consistent individual differences in behavioral tendencies (animal personality) predict the strength and direction of the mate choice component of premating isolation in this system.

**Results:**

We characterized focal females for their personality and found behavioral measures of ‘novel object exploration’, ‘boldness’ and ‘activity in an unknown area’ to be highly repeatable. Furthermore, the interaction term between our measures of exploration and boldness affected focal females’ strength of preference (SOP) for the resident male phenotype in dichotomous association preference tests. High exploration tendencies were coupled with stronger SOPs for resident over alien mating partners in bold, but not shy, females. Shy and/or little explorative females had an increased likelihood of preferring the non-resident phenotype and thus, are more likely to contribute to rare population hybridization. When we offered large vs. small conspecific stimulus males instead, less explorative females showed stronger preferences for large male body size. However, this effect disappeared when the size difference between the stimulus males was small.

**Conclusions:**

Our results suggest that personality affects female mate choice in a very nuanced fashion. Hence, population differences in the distribution of personality types could be facilitating or impeding reproductive isolation between diverging populations depending on the study system and the male trait(s) upon which females base their mating decisions, respectively.

**Electronic supplementary material:**

The online version of this article (doi:10.1186/s12862-016-0712-2) contains supplementary material, which is available to authorized users.

## Background

Animal personality (AP)—also referred to as ‘temperament’ [1]—describes individual differences in behavioral tendencies that are consistent across time and contexts [1, 2]. As a major component of intraspecific phenotypic variation that integrates genomic and environmentally-induced variation [3–5], AP was hypothesized to play a role in various evolutionary and ecological processes [1, 6–9]. For example, previous studies described links between AP and life-history parameters [10, 11], individual space use [12, 13] and dispersal tendencies [14–20]. Moreover, the composition of behavioral types in social groups plays a vital role for the evolution of sociality and cooperative behavior [21–24], and variation in host personalities alters parasite-host interactions, with implications for coevolutionary dynamics [25, 26].

Despite an upsurge of theoretical studies describing potential links between AP and evolutionary processes [7], a recent article highlighted that *“…researchers have almost entirely overlooked potential links between personality traits and speciation*” [27]. For example, no empirical study to date has addressed the potential role of AP in determining the strength and direction of premating isolation during ecological speciation (i.e., speciation during which reproductive isolation is the result of ecologically-based divergent selection; [28]). Our present study examined parapatrically evolving (i.e. diversifying), locally-adapted populations in the species complex of the neotropical freshwater fish *Poecilia mexicana* [29, 30]. We asked whether variation in female preferences for resident over alien mating partners is just due to random noise or if individuals differ predictably in their strength of preference (SOP). We argue that individuals with low SOP-values (e.g., weak discrimination in favor of their own ecotype) and especially those showing a preference for alien male phenotypes are more likely to contribute to mismatched mating and potential hybridization. Links between animal personality and aspects of sexual selection are clearly under-studied [31]. Most studies dealing with this topic focused on female preferences for male behavioral traits (i.e. personality traits of stimulus males) or assortative/disassortative mating based on personality traits [31], while studies on the potential impact of the choosing individuals’ personality on their preferences for mating partners from their own versus an alien ecotype have not yet been conducted.

In this context, AP could play an important role in predicting variation in SOP, and we propose the following mechanistic link: mate evaluation and mating decisions are based on both private sampling and social information use [32–35]. Interestingly, in some species AP predicts the propensity to use private information (obtained from personal sampling) versus social information (obtained from the observation of other individuals; [33, 36, 37]). Individuals with a higher exploration tendency towards a novel object (which can be interpreted as exploration, boldness, or a mix of both, depending on the novel object, the context, and the test species) relied more on private information than less explorative/shy ones [38, 39]. Individuals that rely more on private information should be more experienced in acquiring and using private information and, thus, should cope better with situations in which social information is not available. Less explorative and/or shy individuals, on the other hand, could contribute more to mismatched mating when social information is not available. In our present study, single focal females could chose between a resident male from their own locally-adapted population and an alien male from a different population. As social information use was impossible, we predicted explorative and/or bold females to exhibit stronger SOP for resident over alien male phenotypes than less explorative and/or shyer ones (prediction 1).

Our second hypothesis addresses the question of a potential consistency in choosiness. AP might predict individual choosiness—a trait that is known to vary substantially within and among populations [40–42]—across different mate choice situations. We tested this idea by giving each individual focal female a choice not only between males from their own vs. an alien population (see above), but also between large and small males from their own population. Females of our study species display a strong preference for large male body size along with pronounced variation in females’ SOP [43–45]. We predicted a correlation between individual SOP-values from both mate choice tests (prediction 2).

We examined these hypotheses in a system of locally-adapted populations in the *P. mexicana-*species complex that have repeatedly colonized springs containing toxic hydrogen sulfide (H_2_S) in at least four parallel river drainages in Southern Mexico [46]. Local adaptation to sulfidic conditions involves the evolution of a less H_2_S-susceptible cytochrome-c oxidase (COX) variant in some populations [47], increased constitutive expression of the sulfide:quinone oxidoreductase (SQR; [48]), and parallel morphological changes [29, 49]. For example, larger head sizes of sulfide adapted fish allow for a more efficient oxygen acquisition under sulfidic, hypoxic conditions [49]. Local adaptation in this system is accompanied by varying degrees of reproductive isolation, as revealed by reduced gene flow along the sulfide/non-sulfide interface [50, 51]. Both natural selection against migrants and sexual selection—especially discrimination against alien male phenotypes in females from non-sulfidic habitats—maintain reproductive isolation (reviewed in [46]).

**Fig. 1:**
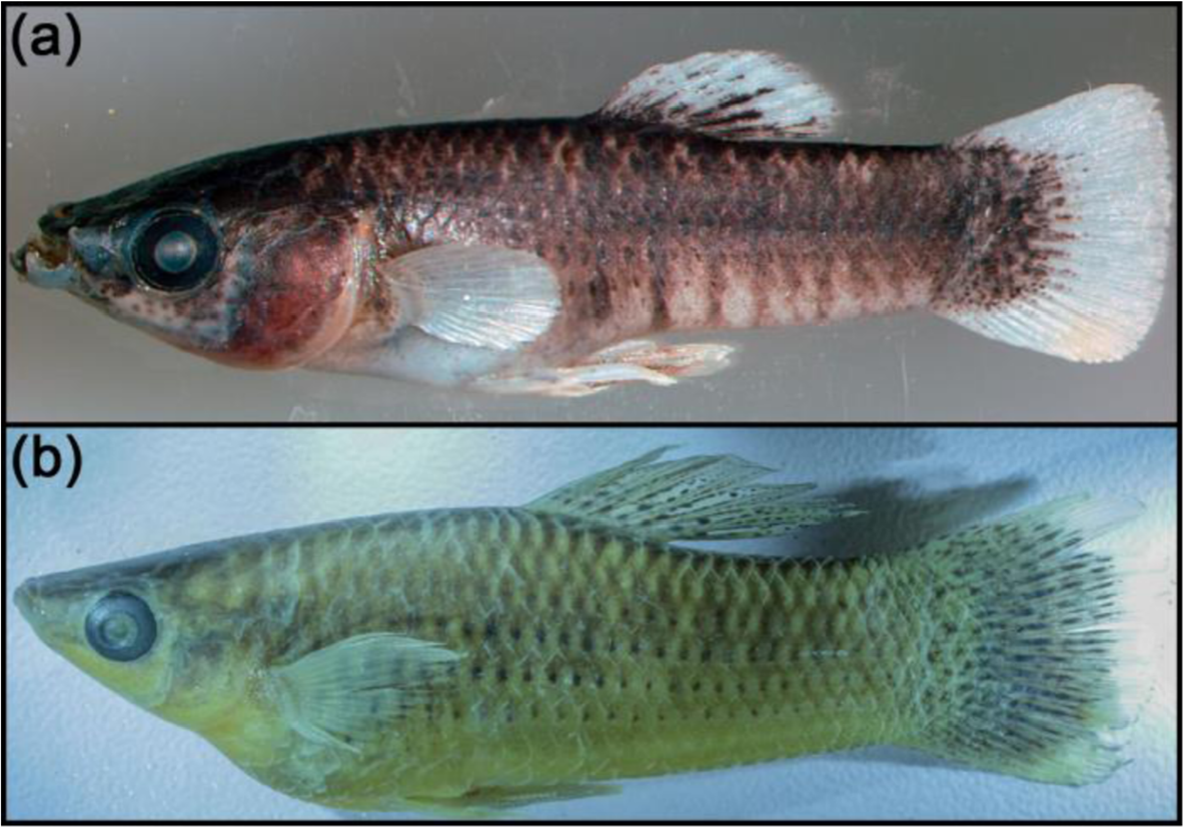
Representative pictures of (**a**) a male sulphur molly, *Poecilia sulphuraria*, and (**b**) a male Atlantic molly, *Poecilia mexicana*, demonstrating the differences in body shape and body pigmentation; this particular male *P. sulphuraria* also possesses lower lip appendages, which are common for the species [46]. Please note, pictures are not on the same scale. Photos by R. Riesch

We studied both hypotheses related to the occurrence of individual variation in female mating preferences in *P. mexicana* inhabiting the Río Pichucalco drainage (Additional file 1: Figure S1), in which sulfide-adapted populations have been described as a distinct species, *Poecilia sulphuraria* ([29, 52]; see Fig. 1 for morphological differences between males from sulfidic and non-sulfidic sites). We characterized individual wild-caught *P. mexicana* females for the personality traits ‘novel object exploration’, ‘boldness’ (measured via ‘freezing time’ after a simulated aerial attack) and ‘activity in an unknown area’. We then tested whether SOP for conspecific males is dependent on focal females’ personality traits, and whether females’ SOP varies consistently across the two different mate choice situations (own vs. alien male phenotypes and large vs. small males).

## Methods

### Test subjects and their maintenance

Test fish were caught with seines (3 mm mesh size) in the Río Pichulcalco drainage between 2nd and 10th of April 2015. We collected *P. sulphuraria* males (*n* = 42) in a sulfidic creek at the Baños del Azufre, while *P. mexicana* stemmed from a nearby freshwater site (*n* = 25 females, *n* = 48 males; Additional file 1: Figure S1). We transferred the fish in aerated coolers within 15 min to the nearby research station (*Centro de Investigación e Innovación para la Enseñanza y el Aprendizaje*) in the city of Teapa, where all behavioral tests were conducted. We maintained all test fish separated by species and sex in small groups of 5 – 6 individuals in plastic containers (52 × 24 × 30 cm; 17 l), equipped with an air pump and filled with water from the respective sampling sites, at ambient temperature. Containers were covered on the outer sides with black plastic foil to minimize disturbance. Fish were fed once a day with commercially available flake food (TetraMin®). To maintain water quality, we exchanged half of the water every two days, for which we used non-sulfidic stream water, such that the *P. sulphuraria* males could gradually adapt to non-sulfidic water conditions, to which they were exposed during the subsequent mate choice tests (see [51]). Using this approach, we prevented abnormal behavior of the *P. sulphuraria* stimulus males (i.e. reduced swimming performance) that might have resulted from an abrupt transition to non-sulfidic conditions.

**Fig. 2:**
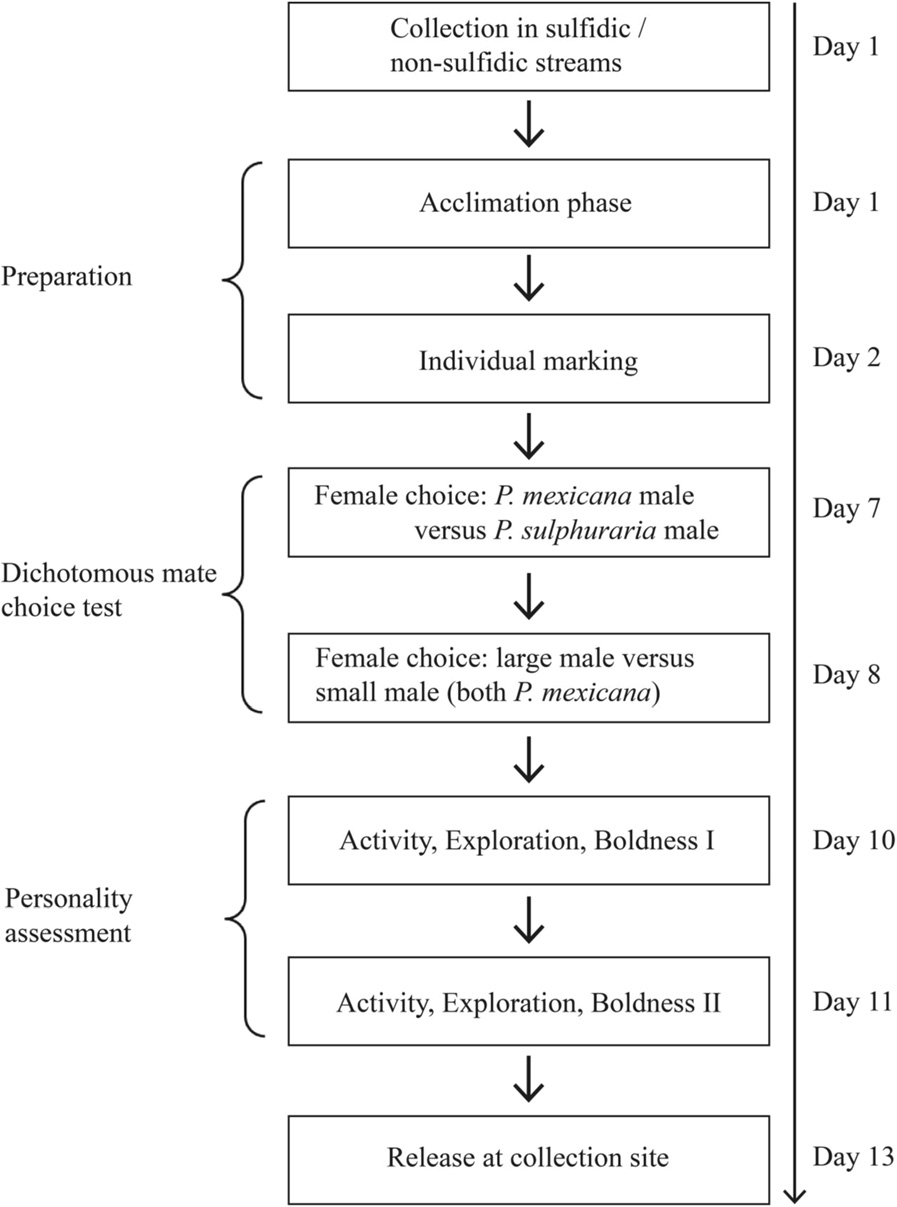
Experimental timeline for the mate choice tests and personality assessments

After an initial acclimatization period of 24 h, all focal females (*P. mexicana*) were marked to allow individual identification. To this end, we briefly anesthetized the females by transferring them into a bucket filled with water from the maintenance tanks and adding a small amount of clove oil (1:10 mixed with EtOH) to the water. Following the protocol described by Croft et al. [53], we injected small spots of visible implant elastomer (VIE, Northwest Marine Technology, Inc.) under the dorsal epidermis (see also [54]). Thus, each individual was given a unique identification tag, enabling us to recognize individuals throughout the behavioral assessments. No mortality was associated with the tagging procedure, and all fish behaved calmly and showed no signs of distress after recovery from anesthesia. After tagging, females were given four days to recover from the mild handling stress. Focal females passed through a series of different behavioral tests; after each test they were given 24 h for recovery in their maintenance tanks (for timeline see Fig. 2).

### Mate choice experiments

Dichotomous mate choice tests (tests 1 and 2) were conducted in parallel, by two experimenters, in two identical transparent Plexiglas tanks (42.6 × 16.5 × 30 cm). The tanks were visually divided into three zones: a neutral zone in the middle of the tank and two lateral preference zones (Additional file 1: Figure S2). Stimulus males were presented in two smaller auxiliary tanks (19.5 × 14.5 × 30 cm) placed adjacent to the two shorter sides of the test tank. Hence, the focal female could choose to associate with the two different male phenotypes on the basis of visual cues, including morphological and behavioral differences. Previous studies demonstrated the importance of visual cues during mate choice of *P. mexicana* [43], while chemical cues play a minor role during mate choice in *P. latipinna* [55]. Association time in this experimental situation has been demonstrated to be a good indicator of female mating preferences in closely related species [56–58].

A webcam (Microsoft LifeCam VX-2000™) was installed centrally above the test tank at approximately 1.5 m height, allowing us to remotely observe the focal female’s movement. Before each trial, we introduced one stimulus male into each of the two auxiliary tanks. Once the males were swimming calmly, we introduced the focal female into a transparent Plexiglas cylinder (10 cm diameter) in the center of the neutral zone and left it undisturbed for three minutes (habituation phase). During this time, focal females could inspect both stimulus males, as the relatively small dimensions of the test tank allowed the female to see both auxiliary tanks from inside the acclimatization cylinder. After the 3-min habituation phase, we gently lifted the cylinder and once the focal female started to swim freely in the water column, we started recording its behavior. We measured times spent in both preference zones during a 5-min observation period as an estimate of the female’s preference for both male phenotypes [51, 56–58]. To detect side biases, we interchanged both stimulus males immediately after the first trial and repeated measurement of association preferences. We gave the focal female two more minutes for acclimatization before the second part of a preference test was initiated. Side bias was assumed if females spent >80 % of the complete 10 min in the same association zone; however, no side bias was detected. Once a trial was completed, we determined the stimulus males’ standard lengths (SL). We made an attempt to use different males as stimulus males for each trial but had to reuse some *P. mexicana* males (two males in test 1 and half of the males in test 2) to create a sufficient number of appropriate stimulus pairs; no male was reused more than once though, and all reused males were used in different dyadic combinations.

We tested each focal female in two different mate choice tests: in test situation 1, females could choose between a conspecific (mean ± SE SL: 36.5 ± 1.2 mm) and a size-matched heterospecific male (*P. sulphuraria*; 35.1 ± 1.0 mm; Mann–Whitney *U* test: *Z* = 1.10, *p* = 0.27, *n* = 25; stimulus males were paired such that the size difference never exceeded 3 mm). In test situation 2, focal females could choose between two different-sized conspecific males (large: 55.6 ± 2.0 mm; small: 43.3 ± 2.0 mm; Mann–Whitney *U* test: *Z* = 3.68, *p* < 0.001, *n* = 25; minimum size difference 9 mm). We summed association times near both stimulus males from the two parts of a trial (before and after switching of side assignments) and calculated the strength of preference (SOP) for both test situations as:

Test situation 1: (time spent with *P. mexicana* male – time spent with *P. sulphuraria* male)/time spent with both males;

Test situation 2: (time spent with large *P. mexicana* male – time spent with small *P. mexicana* male)/time spent with both males.

Thus, SOP-values could range from +1 (maximum preference for the conspecific or large male) to -1 (preference for the heterospecific or small male).

### Personality assessment

In test situation 3, we characterized each focal female along three personality axes: *exploration* as the behavioral response to a novel object, *boldness* as the response to a simulated aerial predator attack and *activity* in an open field test; all tests were performed consecutively in the same tank to minimize handling stress. Our experimental design followed previous studies on poeciliid fishes (*activity*: [34, 59]; *boldness*: [34, 60]). The test arena consisted of a transparent plastic container (52 × 24 × 30 cm) that was placed on grey cardboard and filled with aerated stream water to a height of 15 cm. All outer sides were covered with black plastic foil to minimize disturbance. A grid (5 cm squares) was drawn on the bottom, and three additional marks divided the tank into three equal-sized zones along its long side (Additional file 1: Figure S3). A webcam (Microsoft LifeCam VX-2000™) was installed centrally above the arena (see mate choice tests).

To initiate a trial, we placed the focal female into a Plexiglas cylinder in the center of the test arena. After three minutes for habituation, we gently lifted the cylinder. As *P. mexicana* often freeze for several seconds on the bottom when introduced into a new test arena, measurements started only after the fish resumed swimming (all fish started swimming within 2 min). During a five-min observation period, we counted numbers of crossed squares. We assumed more active fish to cross a larger number of squares, which has been shown to represent a valid personality trait assessment in poeciliids (*P. reticulata*: [59]; *P. latipinna*: [61]; *P. mexicana*: [34]).

After the activity assessment, we retransferred the focal fish back into the Plexiglas cylinder, which we placed close to the wall of one of the small sides of the test arena. We introduced a novel object at the opposite side of the test tank, close to the tank wall. After a brief habituation phase of 1 min, we gently lifted the cylinder and waited until the female resumed swimming. During a five-min observation period we measured the time spent by the female in each of three zones of the tank (Additional file 1: Figure S3). We assigned a rank of 3 to the zone containing the novel object, 2 to the central area, and 1 to the area afar from the novel object and calculated a score expressing individuals’ tendency to explore the novel object as the sum of time [× s^-1^] spent in the three zones, multiplied by the respective rank value. This resulted in high values (max. 900) for explorative and low values (min. 300) for non-explorative individuals. The tendency to explore a novel object can either be interpreted as ‘exploration’ [1], or as ‘boldness’ [62]. We followed the definition by Réale et al. [1] who determined boldness as an individual’s response to risky but not to new situations, the latter being defined as exploration. Still, experiments using the novel object approach could also include a measure of boldness if the novel object or the context in which the novel object is presented elicits frightening responses. We tried to give a clean measure of exploration by using a novel object that was neither completely artificial nor bright in color (the lower half of a transparent bluish plastic bottle filled with pebbles) and thus did not have an intimidating effect on test fish. In a pilot study with laboratory-raised descendants of wild-caught fish of the same species we could not detect any signs of the typical frightening responses towards the novel object (see below).

After the novel object was removed, the female was given three more minutes to recover before a pulley system was used to release a white Ping-Pong ball onto the water surface, simulating an aerial attack (see also [63]). *P. mexicana* females uniformly responded by, first, dashing to a corner and, then, staying on the bottom of the tank, stopping any obvious movements in order to remain inconspicuous (= freezing), before resuming to swim. We terminated a trial when the female was swimming again or after a maximum ceiling value of 300 s. We intended to avoid carry-over effects of the boldness measurement (fish might be intimidated after a simulated predator attack) and ensured that the test arena truly represented an unknown area during the open-field activity test. At the same time, we intended to avoid stress resulting from an additional transfer to another test tank. Therefore, we decided not to randomize the order of the different personality assessments.

After the completion of a trial, females were retransferred into their respective holding tanks and left undisturbed for 24 h before testing was repeated. For the second personality assessment, the novel object was slightly modified (pebbles were replaced by bigger stones and shells) so as to avoid habituation effects. The repeated testing design allowed us to calculate behavioral repeatability [64], a measure of how consistently individuals differed in their behavioral responses. After the last personality assessment, we measured SLs of all focal females upon which all test fish were released at the respective collection sites. All statistical analyses were conducted with the unmodified freezing time values. For display purpose and to ease the discussion, we calculated a ‘boldness score’ as: (300 – freezing time [× s^-1^]), whereby bolder individuals were predicted to resume swimming faster [34].

### Statistical analyses

#### Consistency of personality traits and mating preferences

Our first question was whether focal females would show consistent individual differences in personality traits across both behavioral assessments. Consistency can be inferred from repeatability (*R*)-values of a repeatedly measured trait, defined as:

Variance among individuals/(variance among individuals + variance within individuals) [65].

Following Nakagawa and Schielzeth [64], *R* was calculated from variance estimates obtained from linear mixed models (LMMs) for each personality trait separately. We included no fixed effects (but see Additional file 1: Table S1 for *R*-values obtained from a model in which focal female SL was included as a covariate), as our aim was to provide a conservative measure of within- and among-individual variation [66]. Significant deviations of *R* from zero were tested with likelihood ratio tests (LRT).

Our second hypothesis predicts SOP-values to be consistent across both mate choice situations, and so we asked whether females would show repeatable individual differences in their SOP-values obtained from the two consecutive mate choice experiments. Therefore, we conducted another LMM (similar to those for personality traits) to calculate *R*-values from both mate choice tests, using SOP-values as the dependent variable. We also tested if there is a correlation between SOP-values using Spearman’s *ρ*.

We used SPSS version 23.0 for all statistical analyses. Assumptions of normal error distribution and homoscedasticity were met in case of all dependent variables.

#### Correlations between personality traits

We used arithmetic means from the first and second personality assessment and tested for correlations between our measures of ‘exploration’, ‘freezing time’ and ‘activity’ using Spearman’s *ρ*. We also tested for correlations between the personality traits and ‘focal females’ standard length’. We corrected *α*-levels for multiple testing as *α*’ = 0.05/3 = 0.017.

#### Influence of personality on mate choice decisions

To test whether focal females prefer con- over heterospecific and large over small stimulus males as mating partners, we conducted paired *t*-tests on association times near both types of stimuli in both test situations. The main question of our study was whether and how personality differences among individuals affect females’ mate choice decisions. Since the repeatability (*R*) of SOP-values across both mate choice tests was low and non-significant and we could not find a correlation between SOP-values, we treated SOPs separately in the following analyses. We ran two general linear models (GLMs; one for each mate choice test) using SOP-values as the dependent variable (assumptions of normal error distribution and homoscedasticity were met in case of both dependent variables). We included ‘exploration’, ‘freezing time’, ‘activity’ (in all cases means from both tests), ‘focal females’ standard length’ and ‘size difference between the two stimulus males’ (conspecific – heterospecific and large – small stimulus males, respectively) as covariates in the models. We initially included all two-way interaction terms but step-wise excluded non-significant interactions. Since over-fitting could be a problem in our analyses due to the relatively small sample size (*n* = 25), we reduced the number of factors in the final models by also excluding non-significant main effects. Significant effects did not change qualitatively through this procedure.

As a second step, we ran *post-hoc* non-parametric Spearman rank correlations to estimate the strength of significant main effects and interaction terms (from the final model). Only effects that were significant in the parametric models and for which a strong correlation was uncovered in the *post-hoc* analyses (|*r*_S_| > 0.5) are being discussed in the following (but see Table 1 for the complete models).

**Table 1:** Results of univariate GLMs using SOP-values as the dependent variable. Effects that were retrieved as significant by the GLM and showed |*r*_S_| > 0.5 in *post-hoc* Spearman rank correlations, are highlighted in bold

	*F*	*p*
(a) Con- versus heterospecific male		
Exploration	5.42	0.030
Freezing time	4.74	0.040
**Exploration × freezing time**	**4.53**	**0.045**
(b) Large versus small conspecific male		
Exploration	7.92	0.010
Size difference of focal males	8.80	0.008
**Size female**	**19.33**	**<0.001**
**Exploration × size difference of focal males**	**11.89**	**0.003**

## Results

### Consistency of personality traits and mating preferences

**Fig. 3:**
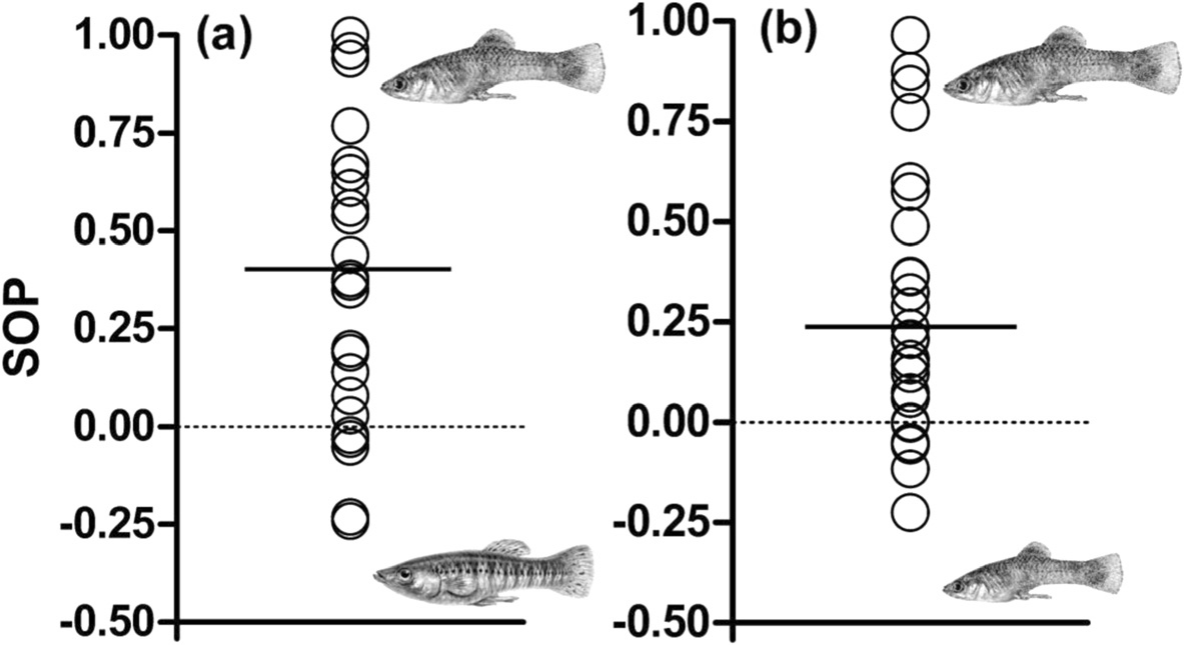
Distribution of individual strength of preference (SOP)-values derived from dichotomous female association preference tests offering (**a**) conspecific versus heterospecific males and (**b**) large versus small males. Solid lines represent the mean SOP across individuals

We found all three personality traits to be highly repeatable: ‘novel object exploration’ (*R* = 0.50, *p* = 0.005, *n* = 25), ‘freezing time’ after a simulated predator attack (our measure of boldness; *R* = 0.64, *p* < 0.001), and ‘activity in an unknown area’ (*R* = 0.52, *p* = 0.003; see Additional file 1: Table S2 for corresponding variance parameters and confidence intervals). As predicted, focal females spent significantly more time near conspecific (mean ± SE: 388.48 ± 25.33 s) than heterospecific stimulus males (156.6 ± 20.88 s) in the first mate choice test (*t*_24_ = 5.09, *p* < 0.001). SOP-values for conspecific stimulus males ranged from -0.24 to 1.00 (mean ± SE: 0.41 ± 0.08; Fig. 3a). Focal fish also spent more time associating with the larger (356.72 ± 21.15 s) than the smaller *P. mexicana* male (192.60 ± 17.63 s) in the second mate choice test (*t*_24_ = 4.39, *p* < 0.001). SOP-values ranged from -0.22 to 0.97 (mean ± SE: 0.29 ± 0.07; Fig. 3b). Only a small portion of the variance seen in SOP-values between the two mate choice situations could be explained by consistent differences among individuals across both mate choice situations, resulting in a low, non-significant *R*-value (*R* = 0.20, *p* = 0.24, *n* = 25). Also, no correlation between SOP-values of both mate-choice situations could be found (*r*_S_ = 0.21, *p* = 0.32, *n* = 25).

### Correlations between personality traits

We found no correlations between ‘exploration’ and ‘freezing time’ (Spearman rank correlation: *r*_S_ = -0.41, *p* = 0.040, *n* = 25, *α*’ = 0.017), ‘activity’ and ‘exploration’ (*r*_S_ = -0.11, *p* = 0.60), or ‘freezing’ time and ‘activity’ (*r*_S_ = -0.02, *p* = 0.94). A strong negative correlation between ‘activity’ and ‘focal females’ standard length’ was uncovered (*r*_S_ = -0.58, *p* = 0.003), meaning that smaller females swam more than larger ones. No correlation was found between ‘exploration’ and ‘focal females’ standard length’ (*r*_S_ = -0.10, *p* = 0.64) or ‘freezing time’ and ‘focal females’ standard length’ (*r*_S_ = -0.02, *p* = 0.91).

### Influence of personality traits on mate choice decisions

#### Female choice for conspecific males

**Fig. 4:**
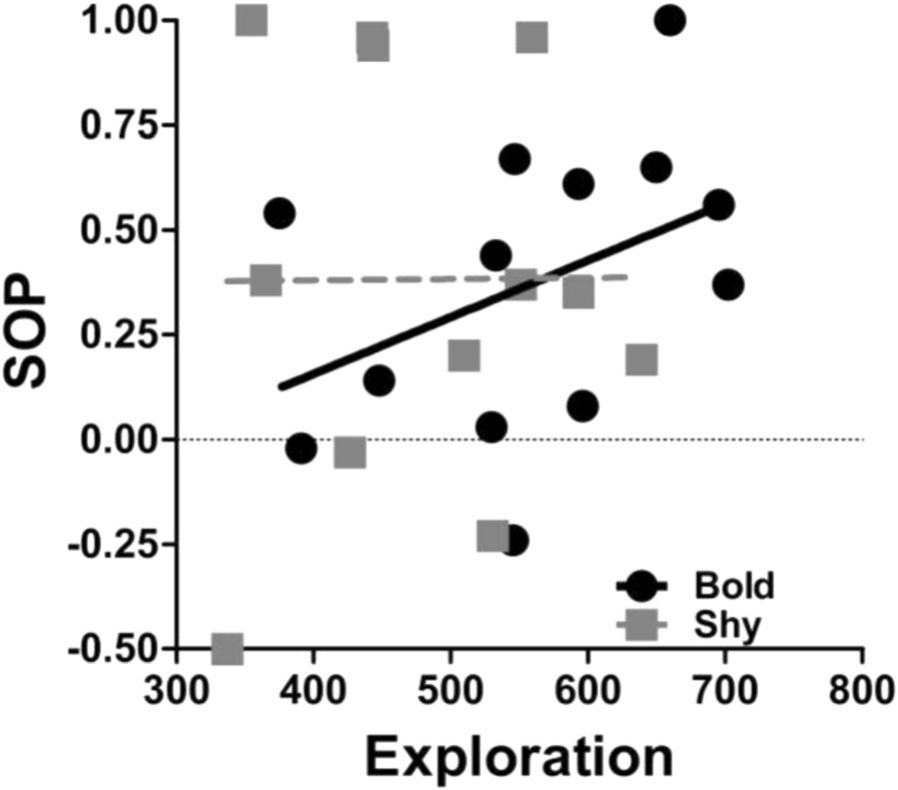
Visualization of the significant interaction term between ‘exploration’ and ‘boldness’ from the univariate GLM using SOP-values for con-over heterospecific males as the dependent variable. SOP-values > 0 indicate preference for con- over heterospecific males

We tested whether personality traits influence females’ SOP for conspecific over heterospecific males. We detected a significant interaction term between ‘exploration’ and ‘freezing time’ (Table 1a). To visualize the interaction effect, we divided focal females into shy (freezing times higher than the empirical mean value of 134.78 s, *n* = 12) and bold (freezing times lower than the empirical mean, *n* = 13). Nonparametric Spearman rank correlations found the SOP to strongly increase with increasing exploration in bold individuals (*r*_S_ = 0.64, *p* = 0.018, *n* = 13) while no such effect was seen in the shyer half of individuals (*r*_S_ = -0.08, *p* = 0.81, *n* = 12; Fig. 4). The two main effects ‘exploration’ and ‘freezing time’ were significant in the final GLM (Table 1a), but had low correlation coefficients in *post-hoc* Spearman rank correlations (exploration: *r*_S_ = 0.24, *p* = 0.25, *n* = 25; freezing time: *r*_S_ = -0.03, *p* = 0.90, *n* = 25).

Neither of the independent variables ‘activity’, ‘female standard length’, nor ‘size difference between the stimulus males’ had statistically significant effects in our initial model (*F*_1,18_ < 3.40, *p* > 0.08) and were thus excluded from the final model.

#### Female choice for large male body size

**Fig. 5:**
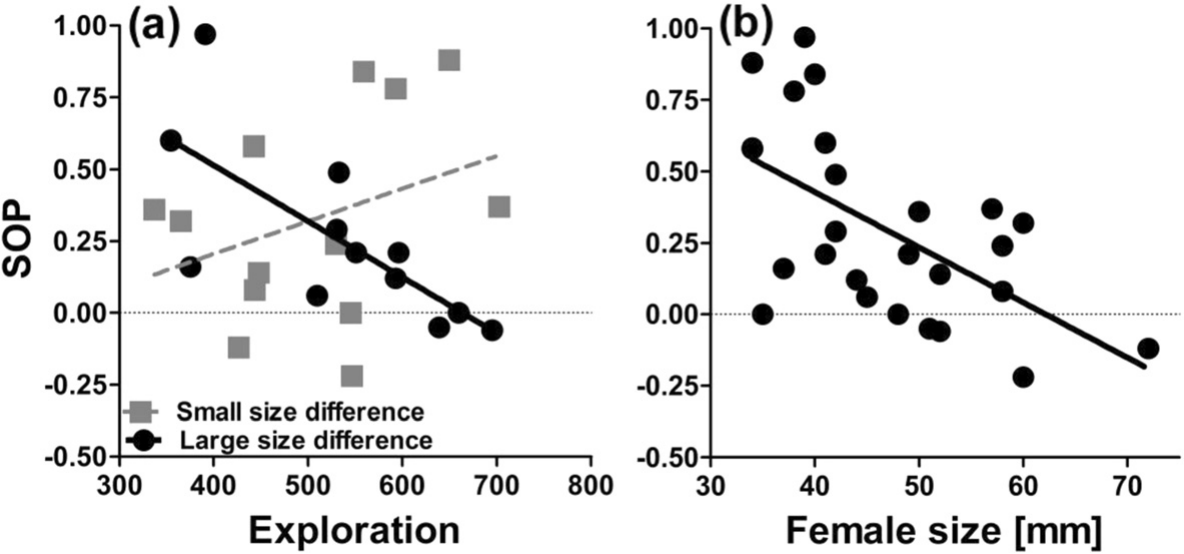
Visualization of significant effects from the univariate GLM using SOP-values for large over small conspecific males as dependent variable. **a** Interaction effect between ‘exploration’ and ‘size difference between the two stimulus males’. **b** Main effect of ‘focal females’ size’. SOP-values > 0 indicate preference for large over small conspecific males

Focal females’ SOP for large male body size was influenced by the interaction effect between ‘exploration’ and ‘size difference between the stimulus males’ (Table 1b). To illustrate the interaction term, we divided the data into two cohorts, for which the size difference between both stimulus males was either smaller (*n* = 13) or larger (*n* = 12) than the empirical mean value of 11.5 mm, respectively. When the size difference was > 11.5 mm, less explorative focal females showed a stronger SOP than more explorative ones (*r*_S_ = -0.71, *p* = 0.01, *n* = 12), while no such effect was seen when the size difference was < 11.5 mm (*r*_S_ = 0.39, *p* = 0.19, *n* = 13; Fig. 5a).

Moreover, the main effect ‘focal females’ body size’ (SL) influenced their SOP for large male body size (Table 1b), and females’ SOP decreased with increasing SL (*r*_S_ = -0.55, *p* = 0.005, *n* = 25; Fig. 5b). Again, the two main effects ‘exploration’ and ‘size difference between the stimulus males’ were retrieved as significant by the GLM (Table 1b) but had low correlation coefficients in *post-hoc* Spearman rank correlations (‘exploration’: *r*_S_ = -0.17, *p* = 0.42, *n* = 25; ‘size difference between stimulus males’: *r*_S_ = -0.22, *p* = 0.30, *n* = 25).

Neither ‘activity’ nor ‘freezing time’ had statistically significant effects and were excluded from the final model (*F*_1,18_ < 0.14, *p* > 0.51).

## Discussion

Ecological speciation describes the process during which reproductive isolation (RI) arises as a consequence of adaptation to ecologically-based divergent selection [28, 67, 68]. Especially in early stages of population divergence, premating isolation plays an important role in determining the strength of RI [28, 69]. In our study system, populations of the *Poecilia mexicana*-species complex have repeatedly adapted to high and sustained concentrations of naturally occurring H_2_S. The independent colonization of sulfidic spring complexes in at least four river drainages not only led to adaptive trait divergence in several character suits associated with oxygen uptake and sulfide detoxification [47–49] as well as offspring survival under toxic conditions [70, 71], but population pairs in each drainage also show emerging RI [51].

Previous studies have investigated mechanisms of premating isolation in our study system: natural selection was found to hamper the migration of individuals into habitat types to which they are not locally adapted, and high mortality was observed especially during the transition from non-sulfidic into sulfidic habitats [50, 51, 72]. In addition, female mate choice plays a role (while males chose their mates rather indiscriminately with respect to different ecotypes): females from non-sulfidic waters showed a strong overall preference against males of the sulfide-adapted ecotype [50, 51, 72], which they may encounter in mixing zones between sulfidic and non-sulfidic stream portions (Additional file 2: Video S1 and Additional file 3: Video S2).

We used this study system to address the question of whether among-individual variance in female preference for males of their own ecotype represents mere statistical noise or whether some females predictably are more likely to contribute to rare population interbreeding. Specifically, we asked if personality traits predict female SOP for resident male phenotypes. In line with this prediction, we found less explorative females to show weaker SOP, even though this effect was markedly stronger in bold than in shy females. We also asked whether females’ SOPs are consistent across mate choice situations. However, we found no correlation between female SOP in two different mate choice situations, namely, discrimination between size-matched own and alien males and discrimination between large and small conspecific males. Moreover, when the size difference between the large and the small stimulus male was pronounced in the latter mate choice situation, less explorative females showed stronger (not weaker) SOP, demonstrating that personality differentially affects female mate choice for resident male phenotypes and mate choice for a sexually selected trait (body size), respectively.

### Effects of personality traits on female preferences for own versus alien males

Less explorative/shy individuals tend to rely more on social information during decision making [38, 39]. Social information, however, is not continuously available, as females cannot always observe other females during their mate choice. Our experimental design did not allow for social information use and so we predicted mate choice of less explorative and/or shy females to be less accurate, resulting in lower (but overall, positive) SOP for resident males. Both hypotheses outlined in the introduction, (1) differences in information use between bold and shy individuals and (2) the potential consistency in choosiness, predict a correlation between SOP values across both mate choice contexts, which we did not find. In the following, we argue that the first hypothesis most likely explains personality-dependent differences in SOP in the first mate choice situation, while direct costs/benefits of associating with certain male phenotypes probably overrode the effect of differential information use in the second experiment (see below).

The interaction between exploration and boldness influenced focal females’ SOP for conspecific males in a way that bolder females showed stronger SOP with increasing exploration tendency. Thus, individuals with a combination of high exploration and boldness showed highest SOPs, while we found a substantial number (20 %) of females with other personality type combinations (shyer than average, less explorative than average, or both) to actually spend more time with the heterospecific stimulus male (Fig. 4). We propose an additional (not mutually exclusive explanation) for this pattern: little explorative and/or shy females could prefer males with a similar personality type. Evidence for fitness benefits arising from assortative mating based on personality traits mostly comes from monogamous species [73–76], but assortative mating also occurs in species in which males only provide sperm [77]. When females of the related guppy (*P. reticulata*) were paired with males that exhibited a similar degree of boldness, they had higher reproductive success than females that were mated with males that differed in boldness scores [78]. Logistic constraints prevented us from assessing personality traits of stimulus males in our present study, but a previous study described that *P. sulphuraria* males are considerably shyer than *P. mexicana* males [79].

Under natural conditions, fishes of both ecotypes indeed co-occur in freshwater and transition zones between sulfidic and non-sulfidic waters where they compete for food (Additional file 2: Video S1) and show some degree of sexual interactions with either species (e.g., precopulatory nipping behavior; Additional file 3: Video S2).

However, hybridization seems to be a rare event in the population-pair studied here, as genetic introgression is low [51]. There are several possible explanations for this finding: most importantly, under natural condition, strong natural selection against migrants (especially in sulfidic waters) in conjunction with sexual selection through female choice effectively restricts gene flow between different, locally adapted populations [50, 72]. Also mate discrimination in extreme environments could be under selection, as hybrids may face a selective disadvantage (reinforcement: [80]). Furthermore, the overall strong preference for conspecific male phenotypes might even be stronger under natural conditions due to the larger average body size of *P. mexicana* males compared with *P. sulphuraria* males (R. Riesch, unpublished data, Additional file 2: Video S1), given that females prefer large-bodied males ([43, 45, 81]; this study).

Our results call for additional experimentation in this and other population pairs. For example, it remains to be studied how variation in population densities and the distribution of personality types within and among populations affect premating isolation. Under low population densities, less explorative and/or shy individuals can barely use social information and so a higher proportion of mismatched mating (and thus, introgression) can be predicted. Nonetheless, we hypothesize that less explorative and/or shy individuals are also less likely to venture into the mixing zones between sulfidic and non-sulfidic stream portions. It will be exciting to elaborate on those aspects in future research projects comparing more population pairs.

### Effects of personality traits on female preferences for large versus small conspecific males

*Poecilia mexicana* females prefer large males as mating partners [43, 45, 81]. In social dominance hierarchies among *P. mexicana* males, the largest male in a group invariably becomes dominant and monopolizes most females [54] and females benefit from mating with dominant males through direct and indirect fitness gains [82, 83]. Smaller males try to compensate for their inferiority in mate competition by showing strongly increased sexual activity [84]. Females try to avoid this sexual harassment, as they suffer considerable fitness costs imposed by sexually harassing males, for example, in the form of reduced feeding opportunities [85, 86]. Staying in the vicinity of larger males protects females from sexual harassment of smaller males ([87]; for *P. latipinna* see [88]).

Still, females show variation in their SOP for large over small males, and we addressed the question of whether some female personality types predictably show weaker SOP for large over small males. We found an interaction between focal females’ exploration tendency and the size difference between both stimulus males. When the size difference was pronounced, less explorative females showed a stronger SOP for large males than explorative females while this effect disappeared when the size difference was less obvious. It remains unclear why less explorative females showed a stronger SOP for larger males than explorative females in this situation. We tentatively argue that more exploratory (potentially more risk-taking) females could be more inclined to accept the costs imposed by sexually harassing (small-bodied) males.

Moreover, we found focal females’ SL to significantly affect SOP, with smaller females showing a stronger SOP for large over small males. This contrasts with other studies reporting on increasing SOP for large over small male body size in larger, more experienced females (*Xiphophorus multilineatus*: [89]; *X. nigrensis*: [90]). While we are lacking an intriguing explanation for this effect, we tentatively argue that a similar explanation as described before can also explain this result: if large-bodied females are more able to escape from male sexual pursuit, then small females should indeed show stronger avoidance of highly harassing, small-bodied males [84]. Differences between the results from our study and the above-mentioned studies on swordtails (*Xiphophorus* spp.) could be due to differences in the degree of male sexual harassment, as numbers of mating behaviors per unit time are much higher in *Poecilia* than in *Xiphophorus* species [91].

## Conclusions

We found evidence that animal personality influences the female mate choice component of premating isolation between locally-adapted populations of the *P. mexicana* species complex. However, the strength and direction in which personality traits affect the SOP for a certain male phenotype depends on the mate choice context and may either impede or facilitate RI. Our present study is amongst the first to address the question of how emerging RI depends on the distribution of personality types in a given population. As such, it leaves open a number of important questions that call for additional experimentation in the future. For example, predation is a major driver of population divergence in other systems (e.g. [92–98]). Increased predation, however, has also been reported to translate into an increased boldness ([79, 99–104], but see [105]). Might differences in predation regimes between repeatedly diverging (convergently evolving) population pairs indirectly affect RI between ecotypes? All else being equal, RI should be higher under elevated predation risk as individuals should, on average, be bolder and more explorative (either through plasticity or selection against shy phenotypes [79, 98, 99]).

Another interesting aspect for future studies in this context is the role of cognitive abilities during mate choice in potential hybridization zones. Personality traits, especially exploration and boldness, were found to correlate with certain cognitive abilities (i.e., the ability to learn a certain task) in a number of species [106–111]. However, the investigation of the relationship between consistent personality traits and cognitive abilities is still at its infancy and poses many methodological challenges [112]. Still, it provides a field of major interest and empirical studies on that topic are desirable. Our study was not designed to test for fishes’ cognitive abilities, however our results do not support a scenario in which personality-correlated consistent individual variation in cognitive abilities alone could explain variation in SOP: first, individuals did not consistently differ in SOP across different mate choice situations; second, personality traits had very different (opposing) effects during the two mate choice situations.

In conclusion, we think that our study provides an interesting new aspect to our understanding of the complexities involved in the early stages of population divergence and speciation. In our system, personality affects female mate choice in a very nuanced fashion, but this need not be the case in other systems. As such, investigations into animal personality traits and their effects on population divergence during incipient speciation are likely to provide new insights into the mechanisms that help promote or constrain further population divergence and ultimately speciation.

## Additional files

Additional file 1: Table S1. Repeatability (R) and significance values for the three behavioral traits used to assess personality differences. R-values are based on variance estimates of separate LMMs including focal females’ body size (SL) as a covariate. p-values were assessed using LR-tests. **Table S2.** Variance parameters obtained from LMMs to calculate repeatabilities for ‘activity’, ‘exploration’ and ‘freezing time’. (a) estimates of within-individual variance and (b) among-individual variance with corresponding 95 % confidence intervals (CI). **Figure S1.** Overview of the study area in Mexico. The magnified section shows the Río Pichucalco with arrows indicating our two sampling sites (1: non-sulfidic site; 2: sulfidic site ‘Baños del Azufre’). Modified from [53]. **Figure S2.** Schematic view of the experimental set-up used in the mate choice trials. The central tank was visually divided into a neutral (NZ, center) and two lateral preference zones (PZ). Two auxiliary tanks holding the stimulus males [in this case: P. sulphuraria male (left) and P. mexicana male (right)] could be inspected by the focal female. **Figure S3.** Schematic view of the test tank used for the personality assessments (top view). Depicted is the start of the assessment of novel object exploration: the focal female (left) and the novel object (grey circle, right) are placed at opposite sides of the tank. Grid lines served for the assessment of activity, during which numbers of squares crossed within 5 min were counted. During the subsequent assessment of exploration tendencies only the two black lines that divide the tank into 3 zones were considered. Zone 1: weak exploration, zone 2: medium exploration, zone 3: strong exploration. (DOCX 3.58 mb) Additional file 2: Video S1. Food competition between Atlantic mollies (*P. mexicana*) and sulphur mollies (*P. sulphuraria*). The site is a mixing zone between sulfidic and non-sulfidic microhabitats with apparently low sulfide content. Silvery fish with a white abdomen and dark bars on the flanks and caudal peduncle are *P. sulphuraria* while beige individuals are *P. mexicana*. Also note the presence of some *Astyanax aeneus* searching for food between the mollies. Fish were recorded on the 24^th^ of April 2016 with a Canon XF200 at full HD resolution with 50 fps and a polarizing filter attached. (MP4 13.6 mb) Additional file 3: Video S2. Sexual interactions (precopulatory nipping at the female gonopore) between *P. mexicana* and *P. sulphuraria* at the same site seen in Additional file 2: Video S1. Besides females, also silvery males with a white abdomen and dark bar patterns (*P. sulphuraria*) and rather ‘dull’ (beige) males (*P. mexicana*) can be observed. Males have yellowish dorsal and caudal fin margins. Within the first 15 s, a *P. sulphuraria* female is approached twice by two *P. mexicana* males and one *P. sulphuraria* male. Also visible are cichlids (*Cichlasoma salvini* and *Vieja bifasciata*) and *Astyanax aeneus*. Fish were recorded on the 24^th^ of April 2016 with a Canon XF200 at full HD resolution with 50 fps and a polarizing filter attached. (MP4 16 mb)
