# Supplementary material for: Does personality affect premating isolation between locally-adapted populations?
Source: BMC Evol Biol. 2016 Jun 23;16:138. doi: 10.1186/s12862-016-0712-2 (PMC4918032; doi:10.1186/s12862-016-0712-2)
Supplement: Additional file 1: Table S1. — Repeatability (R) and significance values for the three behavioral traits used to assess personality differences. R-values are based on variance estimates of separate LMMs including focal females’ body size (SL) as a covariate. p-values were assessed using LR-tests. Table S2. Variance parameters obtained from LMMs to calculate repeatabilities for ‘activity’, ‘exploration’ and ‘freezing time’. (a) estimates of within-individual variance and (b) among-individual variance with corresponding 95 % confidence intervals (CI). Figure S1. Overview of the study area in Mexico. The magnified section shows the Río Pichucalco with arrows indicating our two sampling sites (1: non-sulfidic site; 2: sulfidic site ‘Baños del Azufre’). Modified from [53]. Figure S2. Schematic view of the experimental set-up used in the mate choice trials. The central tank was visually divided into a neutral (NZ, center) and two lateral preference zones (PZ). Two auxiliary tanks holding the stimulus males [in this case: P. sulphuraria male (left) and P. mexicana male (right)] could be inspected by the focal female. Figure S3. Schematic view of the test tank used for the personality assessments (top view). Depicted is the start of the assessment of novel object exploration: the focal female (left) and the novel object (grey circle, right) are placed at opposite sides of the tank. Grid lines served for the assessment of activity, during which numbers of squares crossed within 5 min were counted. During the subsequent assessment of exploration tendencies only the two black lines that divide the tank into 3 zones were considered. Zone 1: weak exploration, zone 2: medium exploration, zone 3: strong exploration. (DOCX 3.58 mb) [file 12862_2016_712_MOESM1_ESM.docx]

**Online Supplemental Material**

**Table S1**

Repeatability (*R*) and significance values for the three behavioral traits used to assess personality differences. *R*-values are based on variance estimates of separate LMMs including focal females’ body size (SL) as a covariate. *p*-values were assessed using LR-tests.

| **Behavioral trait** | **Repeatability (*R*)** | ***p*** |
| --- | --- | --- |
| Activity | 0.39 | 0.030 |
| Exploration | 0.51 | 0.004 |
| Freezing time | 0.65 | < 0.001 |

**Table S2**

Variance parameters obtained from LMMs to calculate repeatabilities for ‘activity’, ‘exploration’ and ‘freezing time’. (*a*) estimates of within-individual variance and (*b*) among-individual variance with corresponding 95% confidence intervals (CI).

| (*a*)  **Behavioral trait** | **Within-individual variance** | **Lower CI** | **Upper CI** |
| --- | --- | --- | --- |
| Activity | 2477.02 | 1422.90 | 4312.07 |
| Exploration | 7977.14 | 4582.38 | 13886.84 |
| Freezing time | 3987.70 | 2290.69 | 6941.90 |
| (*b*)  **Behavioral trait** | **Among-individual variance** | **Lower CI** | **Upper CI** |
| Activity | 2630.52 | 1099.69 | 6292.35 |
| Exploration | 7821.97 | 3180.64 | 19236.15 |
| Freezing time | 7098.28 | 3382.42 | 14896.28 |

**
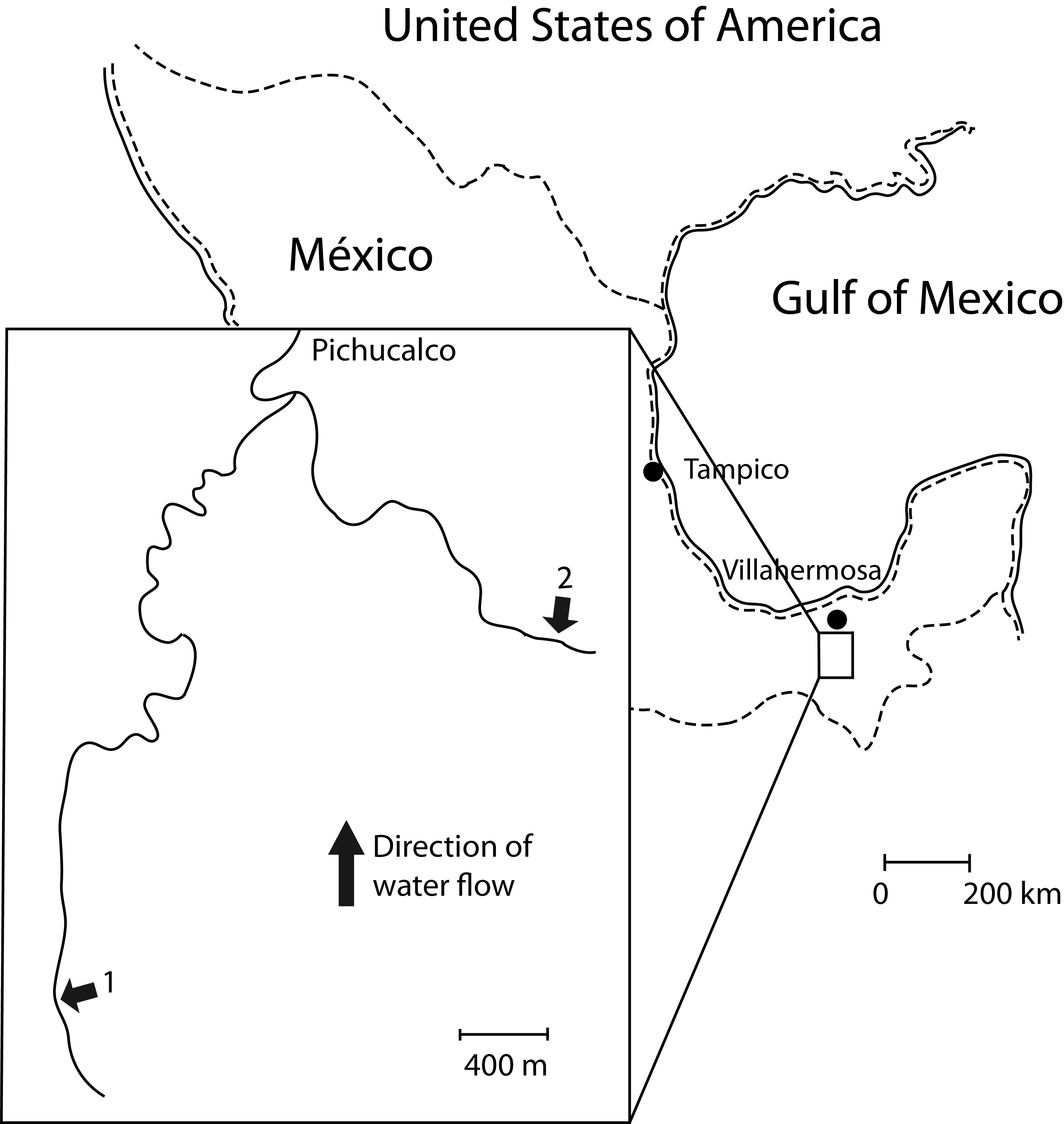
**

**Figure S1**

Overview of the study area in Mexico. The magnified section shows the Río Pichucalco with arrows indicating our two sampling sites (1: non-sulfidic site; 2: sulfidic site ‘Baños del Azufre’). Modified from [53].

**
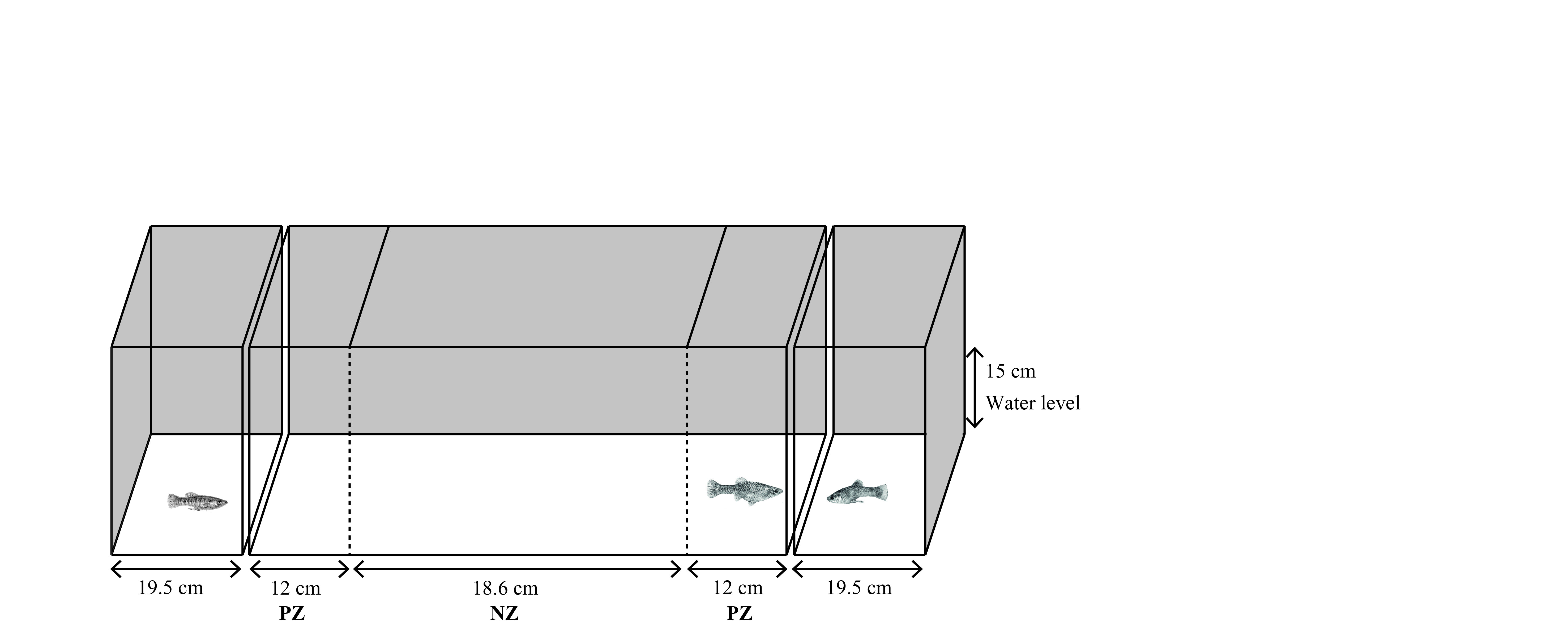
Figure S2**

Schematic view of the experimental set-up used in the mate choice trials. The central tank was visually divided into a neutral (NZ, center) and two lateral preference zones (PZ). Two auxiliary tanks holding the stimulus males [in this case: *P. sulphuraria* male (left) and *P. mexicana* male (right)] could be inspected by the focal female.


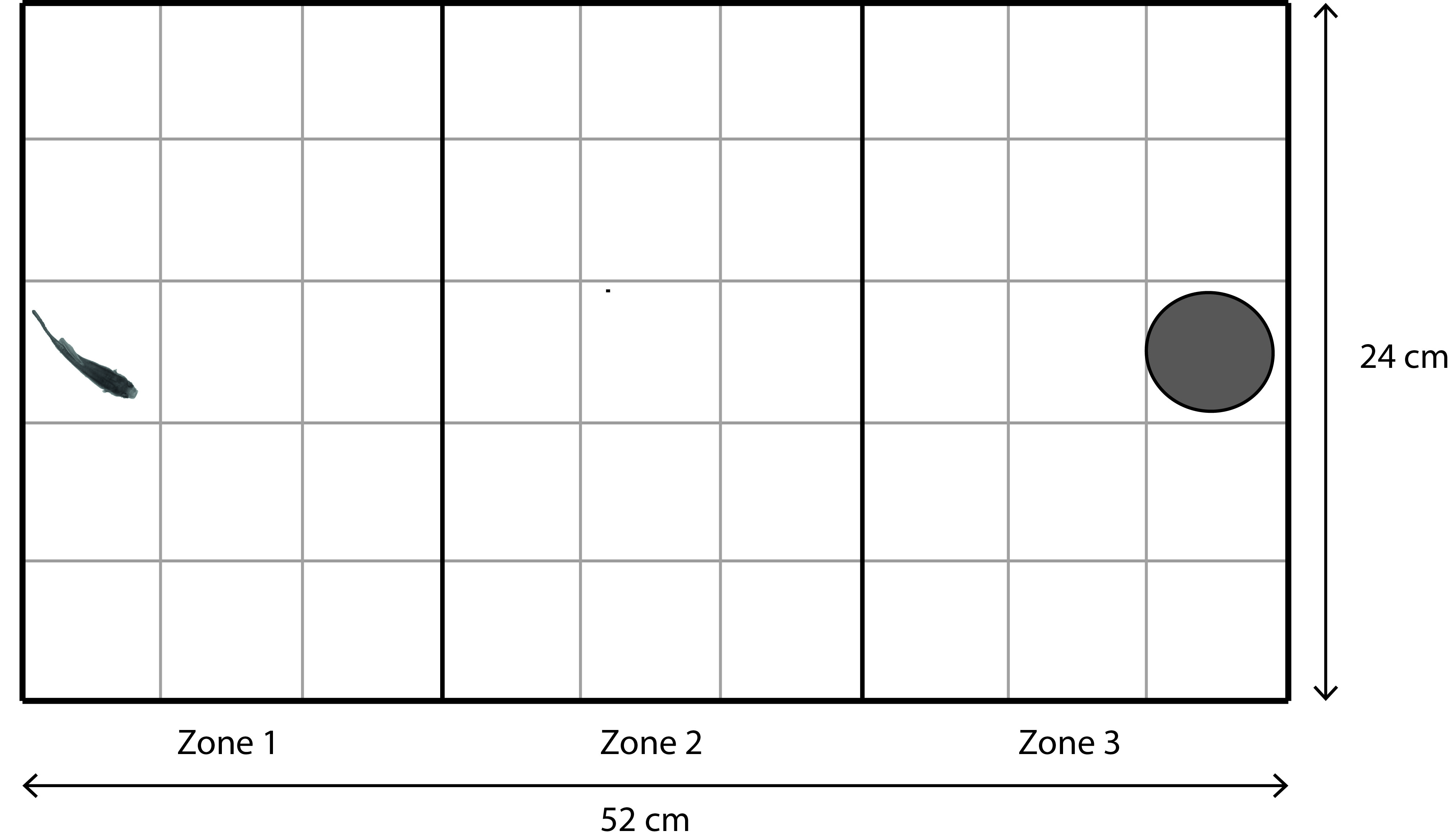


**Figure S3**

Schematic view of the test tank used for the personality assessments (top view). Depicted is the start of the assessment of novel object exploration: the focal female (left) and the novel object (grey circle, right) are placed at opposite sides of the tank. Grid lines served for the assessment of activity, during which numbers of squares crossed within 5 min were counted. During the subsequent assessment of exploration tendencies only the two black lines that divide the tank into 3 zones were considered. Zone 1: weak exploration, zone 2: medium exploration, zone 3: strong exploration.
